# Estrogen Treatment Lowers the Risk of Complications in Menopausal Women with Mild Burn Injury

**DOI:** 10.3390/medicina61020300

**Published:** 2025-02-09

**Authors:** Juquan Song, George Golovko, Kostiantyn Botnar, Amina El Ayadi, Kathleen L. Vincent, Steven E. Wolf

**Affiliations:** 1 Department of Surgery, University of Texas Medical Branch, Galveston, TX 77555, USA; amelayad@utmb.edu (A.E.A.); klvincen@utmb.edu (S.E.W.); 2 Department of Pharmacology, University of Texas Medical Branch, Galveston, TX 77555, USA; gegolovk@utmb.edu (G.G.); kobotnar@utmb.edu (K.B.); 3 Department of Obstetrics and Gynecology, University of Texas Medical Branch, Galveston, TX 77555, USA; swolf@utmb.edu

**Keywords:** female hormone treatment, postmenopausal women, thermal injury, average treatment effect (ATE), retrospective study

## Abstract

*Background and Objectives*: Postmenopausal women are often treated with exogenous female hormones to alleviate physical symptoms and support mental health. We posit that women treated with estrogen fare better following burn injury. *Materials and Methods*: De-identified patient data were obtained from TriNetX, a global healthcare research network. Adult postmenopausal women were enrolled if they were diagnosed with burn injury within 10 years after menopause onset. Patients with pre-existing abnormal uterine bleeding, gynecologic cancer, and chronic liver or heart disease were excluded. The population was grouped into those who received and those who did not receive estrogen treatment (ET) for evaluation of subsequent complications. Cohort balancing was performed using the exact match approach of Inverse Probability Treatment Weighting (IPTW). The average treatment effects (ATEs) and confidence intervals were computed for these balanced cohorts. *Results*: Postmenopausal women with ET had a lower risk of endometrial hyperplasia and malignancy 3 months (ATE = −0.005, −0.006) and 3 years (−0.007, −0.008) after mild burn injury (less than 20% of total body surface area) (*p* < 0.05), regardless of age. At the 3-month timepoint, postmenopausal women aged 45–65 with ET exhibited preventive effects against acute kidney injury (−0.0332), cerebral infarction (−0.0279), breast cancer (−0.0278) and severe sepsis (−0.011) after mild burn injury (*p* < 0.05) compared to women who did not receive ET. After 3 years, 45–65-year-old women with ET exhibited decreased rates of breast cancer (−0.0479) and endometrial hyperplasia (−0.0116) (*p* < 0.05) compared to those without ET. *Conclusions*: Estrogen treatment decreases the risk probabilities of breast cancer and other complications in postmenopausal women from 3 months to 3 years after mild burn injury.

## 1. Introduction

According to the CDC National Center for Health Statistics in 2021, the life expectancy for U.S. men was 73.5 years compared to 79.3 years for women. (https://www.cdc.gov/nchs/data/databriefs/db456.pdf, accessed on 12 December 2024). Aging has become a significant issue as more women survive into old age with a greater incidence of complications and comorbidities [1]. Burn injuries occur in 5 of 100,000 people per year globally [2]. In the United States, about 486,000 burn patients, 32.4% of whom were female, were reported in the American Burn Association (ABA) repository in 2017 [3]. It is well known that both aging and burn injury disrupt the endocrine system with demonstration of adverse events, such as the development of diabetes and bone loss [4,5].

Menopause is an inevitable component of aging and encompasses the loss of ovarian reproductive and endocrine functions [6]. The serum levels of the estrogens estradiol (E2) and estrone (E1) drop from 15–300 pg/mL for premenopausal women to <15 and 7–40 pg/mL in postmenopausal women. The reduction in estrogen contributes to the pathophysiological changes in women [7]. Menopause symptoms include mental health changes and physical activity impairment, and postmenopausal women are treated mostly with exogenous hormone therapy to alleviate these symptoms [8]. Female hormones have also been shown to benefit patients with acute injuries [9]. Both clinical and laboratory investigations suggest that estrogen protects organ function and improves survival after acute injuries [10,11]. Previous studies showed that estrogen protects cardiac function in severely burned mice by reducing mitochondria-derived damage-associated molecular patterns (DAMPs) [12]. Estrogen also protects mice with traumatic brain injury (TBI) from neural inflammation via interleukin-6 (IL-6) activation [13]. We thus posit that women treated with estrogen fare better following severe burn injury.

Burn injury has unfavorable outcomes correlated with age [14]. In the literature, the additional effect of gender is disputed following burn insult, with some studies showing worse outcomes in women [15] and others showing the opposite effect [16]. Postmenopausal women on routine estrogen treatment seem to have improved outcomes. Therefore, this project sought to clarify the role of female exogenous hormones on outcomes after burn injury in postmenopausal women using a large retrospective de-identified patient database.

## 2. Methods

The TriNetX database is a Health Insurance Portability and Accountability Act (HIPAA)- and General Data Protection Regulation (GDPR)-compliant global health research network that provides continuously updated, de-identified electronic medical record (EMR) data, including demographics, diagnoses, procedures, medications and labs. TriNetX, LLC (Cambridge, MA, USA). maintains an Information Security Management System (ISMS). The study was pre-approved by the University of Texas Medical Branch (UTMB) Institutional Review Board (IRB #20-0085). De-identified EMRs were collected from the TriNetX database Research Network in March 2023.

**Figure 1:**
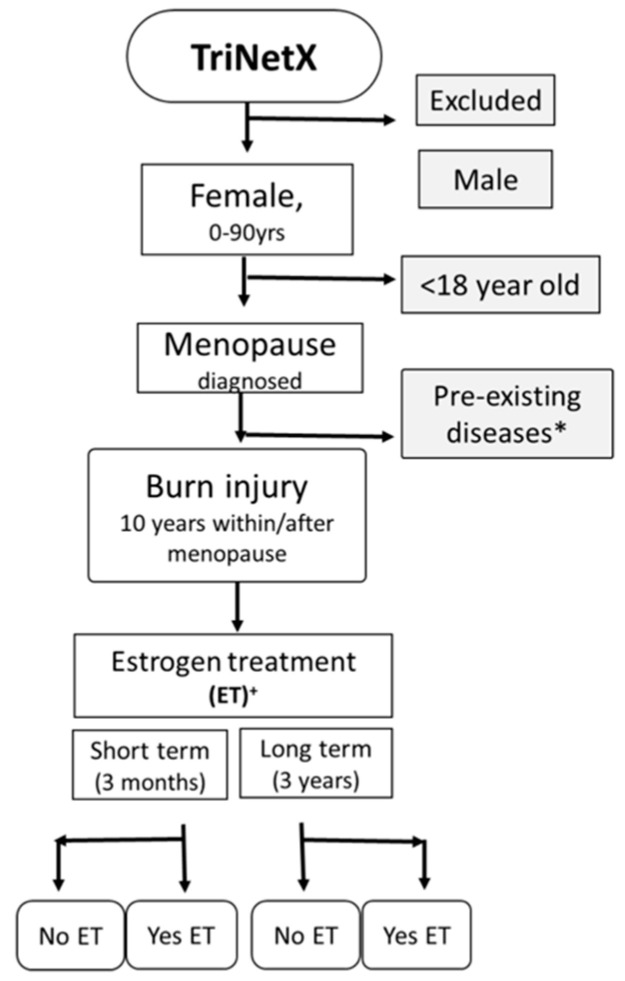
Study population consort flow diagram. Data on menopausal women were collected from the TriNetX research network database. Patients with pre-existing diagnoses of breast cancer; genital organ malignancy; and chronic diseases of the heart, kidney and liver were excluded (*). The populations were sequentially divided into four groups by estrogen treatment (ET) from 6 months before till after injury (+), along with short-term (3-month) and long-term (3-year) effects.

The study included female patients older than 18 years of age with a diagnosis of menopause if they further encountered burn injury within 10 years. Patients with pre-existing abnormal uterine bleeding, gynecologic cancer and chronic liver/heart/kidney diseases were excluded. The study population were stratified into 2 pairs of cohorts, including those who received and those who did not receive estrogen treatment (ET) from 6 months before burn injury until after injury. The consort flowchart of the study is shown in Figure 1. The groups were compared for evaluation of complications in both short- (3-month) and long-term (3-year) periods. The majority of women experience menopause at age 45–55 years [17]. In this study, we also found that the ages of the members of our patient population were mostly within the range of 45–65 years (Supplementary Figure S1). Therefore, we further investigated ET treatment in this age range. The patients and the complications (“progress outcomes”) were identified with ICD-10-CM UMLS codes (the International Classification of Diseases, Tenth Revision, Clinical Modification in the Unified Medical Language System), while the estrogen treatment was identified with the National Drug Code (NDC) (Supplementary Table S1).

We observed estrogen use in 2060 postmenopausal burn-injured women, with 14 known medications recorded (Supplementary Table S2). Estradiol and conjugated estrogens were the two most-prescribed medications at 48.98% and 31.46%, respectively. The other most highly prescribed medications included ethinyl estradiol (3.79%) and norgestimate (2.62%) for birth control, as well as raloxifene (2.09%), an estrogen receptor modulator.

Four primary cohorts were evaluated in this study: short-term (3-month) use of ET vs. no use of ET and long-term (3-year) use of ET vs. no use of ET. Cohort balancing was then performed regarding age, race and ethnicity using an exact match approach and controlled by the chi-square test. The average treatment effects (ATEs) and confidence intervals (CIs) were computed for these harmonized cohorts when the patients were further stratified according to mild (0–19%) and severe (>20%) total body surface area (TBSA) burns. Significance was accepted at *p* < 0.05. All data manipulation was executed using a custom Python (version 3.10) program in conjunction with the SciPy (version 1.11.1) and Pandas (version 2.0.3) libraries.

## 3. Results

Short-term (3-month) complications in postmenopausal burn patients who received estrogen treatment

Demographic descriptions. There were 11,816 patients who did not receive treatment and 2226 patients who received treatment included in the 3-month observational period. The mean ages and standard deviations (SDs) were 60 ± 12 years (Cohort 1, no treatment) and 58 ± 12 years (Cohort 2, estrogen treatment). By comparing the two cohorts, significant differences were observed in Hispanic ethnicity (5.57% in the treatment group vs. 6.88% in the non-treatment group) (*p* < 0.05), White ethnicity (76.73% in the treatment group vs. 68.27% in the non-treatment group) and African American ethnicity (11.14% in the treatment group vs. 17.05% in the non-treatment group) (*p* < 0.05) (Table 1).

**Figure 2:**
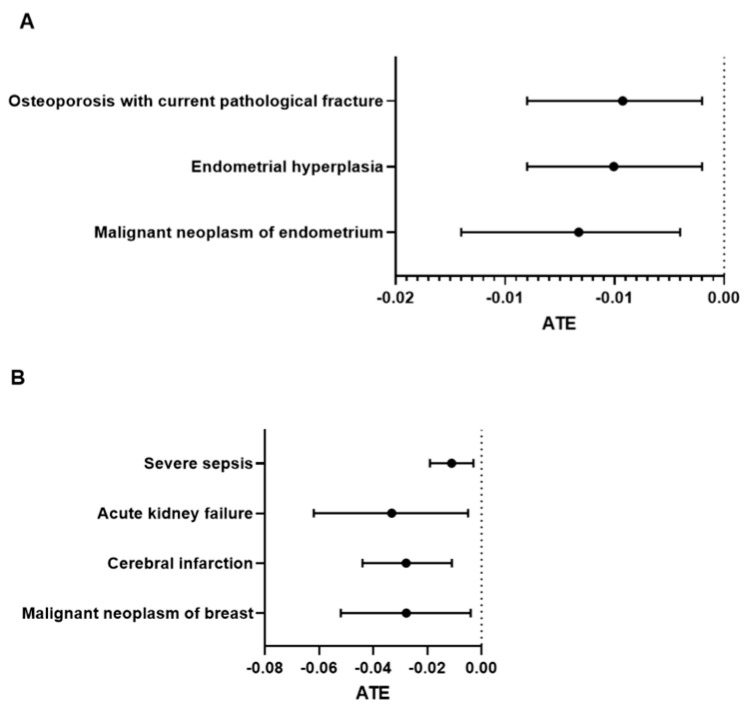
**Progress outcomes of menopausal women with exogenous estrogen treatment, including** (**A**) all adults and (**B**) menopausal women aged 45 to 65 years old after 3 months following mild burn injury. The average treatment effects (ATEs) of diagnoses with 95% confidence intervals (CIs) are presented in forest plots.

Short-term (3-month) complications in adult postmenopausal women. In the 3-month observational period after mild burn injury, women with estrogen treatment had decreased risks of endometrial hyperplasia [ATE = −0.00504, CI: −0.009 to −0.001], malignant neoplasm of the endometrium [ATE = −0.00664, CI: −0.012 to −0.002] and osteoporosis [ATE = −0.00463, CI: −0.009 to −0.001] (*p* < 0.05). No other complications were significantly altered in patients with ET when suffering from either mild or severe burn injury (Figure 2A).

Short-term (3-month) complications in postmenopausal women from 45 to 65 years old. We found significant preventive effects of estrogen treatment in mild burn patients aged 45 to 65 years. The decreased risks, expressed as negative values for ATEs for malignant neoplasm of the breast, cerebral infarction, acute kidney failure and severe sepsis, are noted in Figure 2B (*p* < 0.05).

Other evaluated complications, including death, other unspecified sepsis (streptococcal/streptococcal sepsis), hypertension and wound infection, were not statistically significantly different between the groups.

### Long-Term (3-Year) Complications in Postmenopausal Burn Patients with Estrogen Treatment

Demographic descriptions. There were 12,016 patients aged 60 ± 12 years who did not receive treatment and 3237 patients aged 58 ± 12 years who received estrogen treatment in the 3-year observational period. By comparing the two cohorts, it was observed that there were significant differences in Hispanic ethnicity (5.78% in the treatment group vs. 7.11% in the non-treatment group) (*p* < 0.05), White ethnicity (76.03% in the treatment group vs. 68.44% in the non-treatment group) and African American ethnicity (11.80% in the treatment group vs. 17.19% in the non-treatment group) (*p* < 0.05) (Table 2).

**Figure 3:**
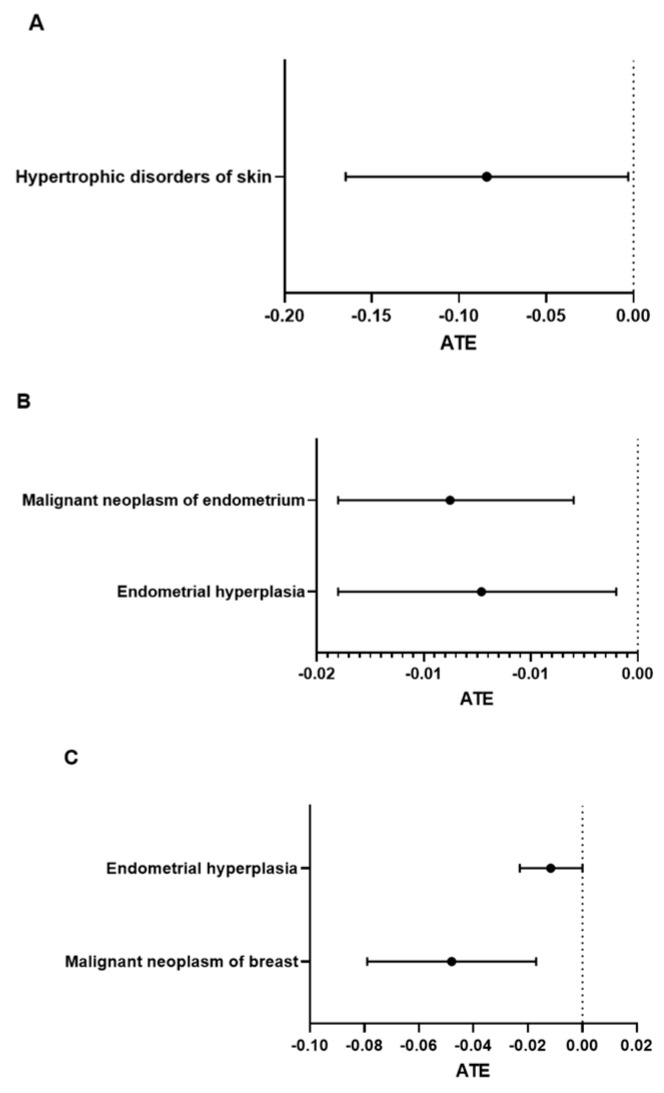
**Progress outcomes of menopausal women with exogenous estrogen treatment 3 years after injury, including** (**A**) all adult patients with severe burn injuries, (**B**) all adult patients with mild burn injuries and (**C**) 45–65-year-old patients with mild burn injuries. The average treatment effects (ATEs) of diagnoses with 95% confidence intervals (CIs) are presented in forest plots.

Long-term (3-year) complications in postmenopausal women with treatment. When observation extended to 3 years after injury, ET treatment was associated with decreased risk of complications in burn-injured women. In severe burn patients, ET treatment had a preventive effect on hypertrophic disorder of the skin [ATE = −0.08407, CI: −0.165 to −0.003] (Figure 3A). In mild burn patients, ET treatment had preventive effects which were similar to those observed in the short term, including endometrial hyperplasia [ATE = −0.0073, CI: −0.014 to −0.001] and malignant neoplasm of the endometrium [ATE = −0.00877, CI: −0.014 to −0.003] (*p* < 0.05). The values of ATEs for the complications are also shown in Figure 3B.

Long-term (3-year) complications in postmenopausal women aged 45 to 65 years. When observation of complications extended to 3 years after injury, patients aged 45 to 65 years who received ET treatment had better outcomes. With estrogen treatment, the ATEs were −0.04798 [CI: −0.079 to −0.017] for malignant neoplasm of the breast and −0.01162 [CI: −0.023 to −0.001] for endometrial hyperplasia (Figure 3C).

The ATEs for other evaluated complications, including death, other unspecified sepsis (streptococcal/streptococcal sepsis) and hypertension, were not statistically significantly different between the groups.

## 4. Discussion

In this study, we observed that fewer Hispanic postmenopausal women received ET and that more White postmenopausal women received ET from 6 months before until after burn injury. Estradiol (49%) and conjugated estrogen (31%) were the 2 most common out of the 14 medications given to patients in this study. Applying the IPTW approach for ATE estimation, we found that patient outcomes were affected by estrogen treatment over 3 months up to 3 years following burn injury. Burn-injured postmenopausal women had a lower incidence of endometrial hyperplasia and malignancy when receiving estrogen therapies. As for the majority population of postmenopausal women, or those aged 45 to 65 years old, treatments were also associated with reductions in breast cancer and other short-term and long-term complications.

Considering the side effects of estrogen treatment, we excluded patients who had gynecological diseases, including abnormal uterine bleeding; female genital organ cancer; and chronic diseases of the kidney, liver and heart from the study. In the estrogen group, this study specifically included postmenopausal women who had been on estrogen treatment from 6 months before their injuries until after the burn injuries to ensure that they had been exposed to estrogen at the time of insult. The estrogen-treated patients had lower incidences of endometrial hyperplasia and malignancy, indicating that the main estrogen effects might shift in burn patients as their systemic status shifts to hypermetabolic and hyperinflammatory states following severe injury. Estrogen did show protective effects against hyperinflammatory responses. Postmenopausal women had lower levels of primary female sex hormones. The endocrinal disturbance could affect the immune and metabolic responses in menopausal women with mild burn injury. Alessandro Villa reported that activation of the intracellular estrogen receptor shortens the LPS-induced pro-inflammatory phase in vitro [18]. Indeed, the 3-month observational results showed estrogen treatment benefits in terms of reduced rates of acute kidney injury (AKI), cerebral infarction and severe sepsis in mild burn patients. In the 3-year long-term observation, estrogen use also showed benefits regarding hypertrophic scarring in severe burn patients with burn sizes greater than 20% TBSA.

Estrogen has been reported to provide a protective function in improving kidney repair [19] and maintaining phosphorus homeostasis in the proximal tubule via its receptors [20]. Clinical observations have shown that females have less risk of AKI development [21] and slower CKD development [22]. Acute renal injury was affected by burn-induced ischemic injury or overburdening of flow in an unstable hemodynamic alteration [23]. The incidence rates of acute kidney failure (AKF) varied up to 30% depending on the study population [24,25]. The incidence of AKF was about 3% in our study; we observed that estrogen treatment was beneficial in reducing AKF after burn injury, suggesting an acute protective effect of estrogen in the kidneys. This benefit was observed in the 45–65-year-old menopausal women only in the 3-month short-term period, further implying its protective effects against burn insult. We also observed a preventive association between estrogen treatment in menopausal women and cerebral infarction in this study. Myocardial dysfunction is characterized by slowed isovolumic relaxation, cerebral infarction and stroke, and estrogen treatment had benefits for postmenopausal women by reducing such effects [26]. Burn induces myocardial dysfunction [27] and ischemic stroke [28], but estrogen may afford cardioprotective effects, which may in turn reduce the risk of cerebral infarction in menopausal women with burn injury.

Wound healing is always a big concern for patient recovery after burn injury. Estrogen promotes healing by modulating the inflammatory response and inhibiting macrophage migration inhibitory factor (MIF) expression in wound sites [29]. Estrogen receptors (ERs) act as ligand-activated transcription factors and regulate target gene expression at the transcriptional level. Though its non-genomic actions are not completely clear, most investigators agree that estrogen triggers intracellular kinases and ions and Ca^2+^ and K^+^ channels [30]. Topical estrogen applications are known to be beneficial for cutaneous wound healing [31]. The most conventional mechanism could be that estrogen increases the late TGF beta signaling pathway and promotes cell proliferation [32]. Systemic and topical estrogen treatment also modulate wound healing by attenuating the inflammatory response with a reduction in macrophage migration inhibitory factor [29]. Another mechanism was also observed through estrogen receptor beta but which did not rely on its anti-inflammatory activities [33]. Estrogen deficiency is detrimental to the wound healing process in postmenopausal women [34]. As expected, we observed that the incidence of hypertrophic scarring decreased in severe burn patients with estrogen treatment at the 3-year timepoint.

While White women comprised the majority of the patients in both groups, they only represented 68% of the non-treated population, which was a significantly lower proportion than in the estrogen treatment group (76%). The second most common ethnic group was African American women, who made up 17% of the untreated group and 11% of the treated group. The patient distributions based on HRT treatment biases were similar to those seen in previous studies going back to 1999 [35]. Brown et al. found in a local retrospective study that among the 8968 women included, there were 23% Latina, 25% African American and 33% White women. Both African American (OR: 0.70; 95% CI: 0.60, 0.81) and Latina women (OR: 0.70; 95% CI: 0.58, 0.84) were less likely to be prescribed HRT [35]. Considering the higher risk of adverse cardiometabolic profiles in Black women in the Study of Women’s Health Across the Nation (SWAN) [36], there should be increased attention to ensuring equitable access to HRT treatment in high-risk populations. Demographics such as race and ethnicity [37], and even age [38], are related to progress in burn injury treatment.

Since estrogen replacement in postmenopausal women is recommended to treat an individual patient’s symptoms, no standard approach was followed to specify the dose, medication combination or route of administration. The effects of estrogen treatment vary based on the length of observation time and unfavorable risks, including ovarian cancer, reported in postmenopausal women after 5-year ET treatment [39]. Estradiol (E2), the form of estrogen produced mainly by the ovaries, was the type most commonly used by the postmenopausal women in this study. We evaluated 3-month and 3-year timepoints after burn injury to focus on the effect of HRT on healing from burn injury. We found general negative ATE scores in burn patients who were taking estrogen treatment, suggestive of an overall beneficial effect. Comparing the data between the two populations (i.e., ET use and non-use), preventive outcomes were generally observed in those who used ET.

Estrogens are commonly combined with progestins, especially in women with an intact uterus; however, we have not estimated the combined effects of estrogen and progestins. Estrogen treatment for women is utilized to compensate for the low level of estrogen after menopause to treat specific symptoms rather than to restore premenopausal levels. However, the varied complications observed in the burn population need to be validated with prospective studies in the future. As AKI is one of the major concerns following burn injury, the benefit for preservation of kidney function has been noted. Meanwhile, the other systemic effects must be considered. Further evaluation of the treatment with established postmenopausal animal models could address the mechanistic background questions [40]. Given that testing of dose-dependent effects in preclinical models is required, we are commencing a pilot study to investigate the effect of 17 beta-estradiol in ovariectomized female rats following burn injury. We hypothesize that estrogen treatment will alleviate endocrine disruption and improve rat recovery following burn injury. Prospective studies are also warranted to further understand the clinical scenario and confirm the use, including the timing and dose, of estrogen in burn-injured postmenopausal women.

We endeavored to specify conjugated estrogen vs. estradiol use while excluding less commonly used or unknown estrogen formulations during the study. However, there were limitations in what information could be obtained from the de-identified sample with respect to medication specifications, such as dose strength, route and duration. The TriNetX database collects data from 61 healthcare organizations in the United States. Due to its regulations to protect patient privacy, patients from specific regions or burn-certified hospitals are not available. Another limitation is that the outcome data were obtained by ICD-10 code identification, which can lack information regarding relevant clinical features, such as medications and procedures related to the outcome. Variability in the recognition and input of ICD codes from different healthcare resources could have introduced bias in this study.

In this study, we used the statistical method of estimating variables’ average treatment effects (ATEs). About 15,000 patients were included for the ATE calculation. For cohort balancing, we used the Inverse Probability Treatment Weighting (IPTW) approach, which assigns different weights for different patients according to the propensity score estimated via logistic regression. Overall, the ATE approach is more general because it includes all patients. However, the method heavily depends on selected confounders, and we are aware that the current observational results need to be further investigated and confirmed with other evidence.

## 5. Conclusions

In conclusion, estrogen treatment in postmenopausal women aged 45–65 years old was associated with decreased acute kidney injury and sepsis 3 months after mild burn injury and with decreased breast malignancy from 30 months to 3 years after both mild and severe burn injury. However, the benefits were not observed in all age ranges and varied between short- and long-term follow-up. These inconsistent observations require further studies to determine the specific dose and duration of estrogen treatment, as well as the effects of its combination with progesterone, to aim to capture the benefits that have been seen in preclinical studies.

## Figures and Tables

**Table 1:** Demographic profiles of postmenopausal women in the 3-month observation period following burn injury.

Group		Cohort 1		Cohort 2		
Treatment		No		Yes		
		Patients	% of Cohort	Patients	% of Cohort	*p*-Value
		11816		2226		
	age [mean (SD)]	60 (12)	100	58 (12)	100	1.0000
Ethnicity						
	Hispanic	813	6.88	124	5.57	0.0260
	Not Hispanic	8887	75.21	1785	80.19	0.0001
	Unknown	2116	17.91	317	14.24	0.0001
Race						
	American Indian or Alaska Native	47	0.40	8	0.36	0.9355
	Asian	258	2.18	37	1.66	0.1355
	African American	2015	17.05	248	11.14	0.0001
	Native Hawaiian or Other Pacific Islander	11	0.09	2	0.09	1.0000
	Unknown	1418	12.00	223	10.02	0.0084
	White	8067	68.27	1708	76.73	0.0001

**Table 2:** Demographic profiles of postmenopausal women in the 3-year observation period following burn injury.

		Cohort 1		Cohort 2		
Treatment		No		Yes		
		Patients	% of Cohort	Patients	% of Cohort	*p*-Value
		12016		3237		
	age [mean (SD)]	60(12)	100	58 (12)	100	1.0000
Ethnicity						
	Hispanic	854	7.11	187	5.78	0.0087
	Not Hispanic	9087	75.62	2558	79.02	0.0001
	Unknown	2075	17.27	492	15.20	0.0057
Race						
	American Indian or Alaska Native	46	0.38	11	0.34	0.8465
	Asian	258	2.15	54	1.67	0.1013
	African American	2066	17.19	382	11.80	0.0001
	Native Hawaiian or Other Pacific Islander	9	0.08	4	0.12	0.6150
	Unknown	1413	11.76	325	10.04	0.0069
	White	8224	68.44	2461	76.03	0.0001

## Data Availability

All relevant data are available within the manuscript. Any material and information generated during the study will be available for sharing with other researchers under appropriate institutional agreements. Any inquiries should be directed to the corresponding author.

## Supplementary Materials

The following supporting information can be downloaded at: https://www.mdpi.com/article/10.3390/medicina61020300/s1, Table S1: Codes applied for patient, medication and disease identifications; Table S2: Patient distribution with medication treatment; Figure S1: Distribution of patients aged 18 to 90 years in Cohort 1 (without medication) and Cohort 2 (with medication).
