# Supplementary material for: Estrogen Treatment Lowers the Risk of Complications in Menopausal Women with Mild Burn Injury
Source: Medicina (Kaunas). 2025 Feb 9;61(2):300. doi: 10.3390/medicina61020300 (PMC11857297; doi:10.3390/medicina61020300)
Supplement: Supplementary file 1 [file medicina-61-00300-s001.zip › medicina-3343630-supplementary.pptx]

## Slide 1
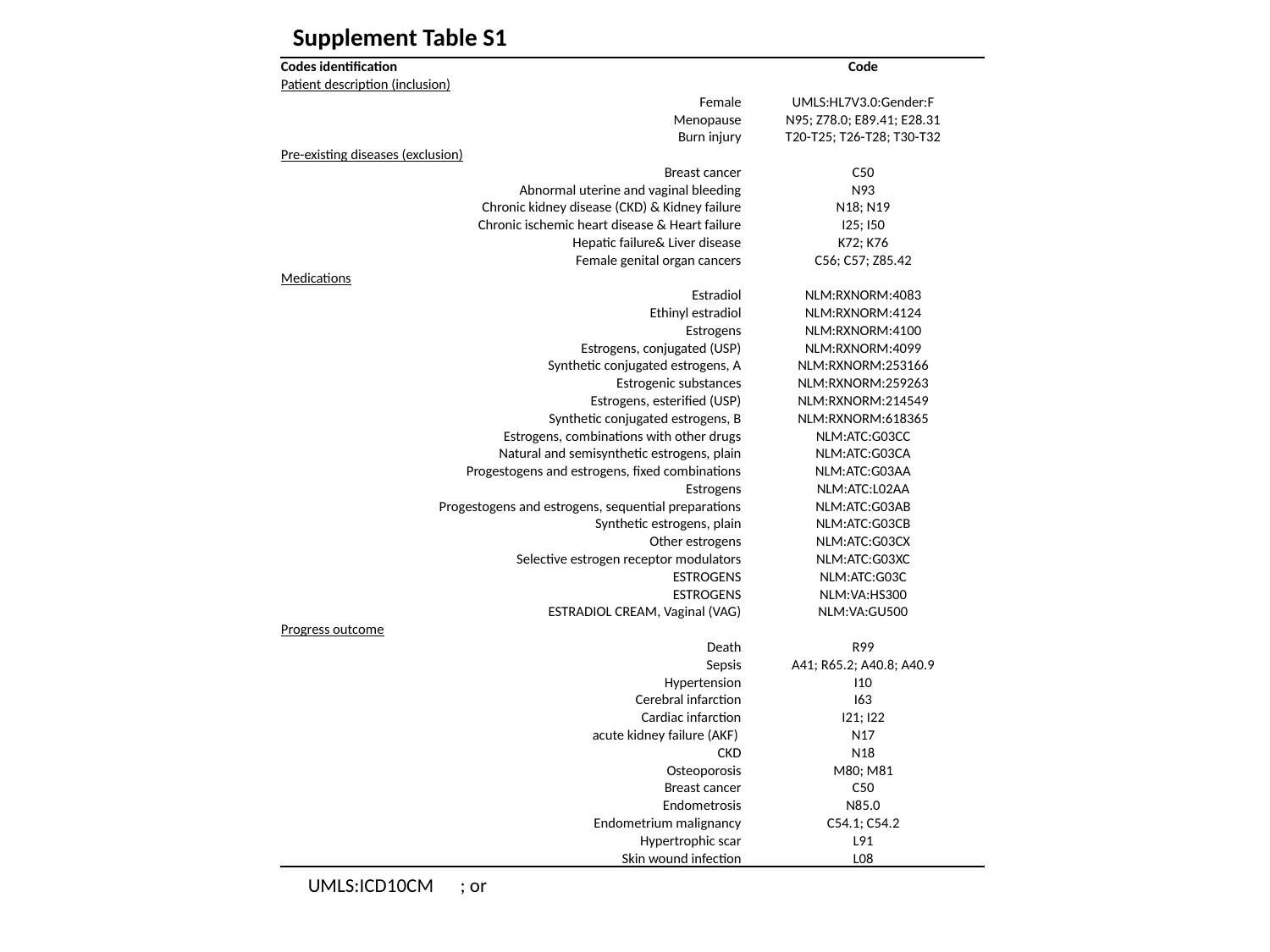

Supplement Table S1
| Codes identification | Code |
| --- | --- |
| Patient description (inclusion) | |
| Female | UMLS:HL7V3.0:Gender:F |
| Menopause | N95; Z78.0; E89.41; E28.31 |
| Burn injury | T20-T25; T26-T28; T30-T32 |
| Pre-existing diseases (exclusion) | |
| Breast cancer | C50 |
| Abnormal uterine and vaginal bleeding | N93 |
| Chronic kidney disease (CKD) & Kidney failure | N18; N19 |
| Chronic ischemic heart disease & Heart failure | I25; I50 |
| Hepatic failure& Liver disease | K72; K76 |
| Female genital organ cancers | C56; C57; Z85.42 |
| Medications | |
| Estradiol | NLM:RXNORM:4083 |
| Ethinyl estradiol | NLM:RXNORM:4124 |
| Estrogens | NLM:RXNORM:4100 |
| Estrogens, conjugated (USP) | NLM:RXNORM:4099 |
| Synthetic conjugated estrogens, A | NLM:RXNORM:253166 |
| Estrogenic substances | NLM:RXNORM:259263 |
| Estrogens, esterified (USP) | NLM:RXNORM:214549 |
| Synthetic conjugated estrogens, B | NLM:RXNORM:618365 |
| Estrogens, combinations with other drugs | NLM:ATC:G03CC |
| Natural and semisynthetic estrogens, plain | NLM:ATC:G03CA |
| Progestogens and estrogens, fixed combinations | NLM:ATC:G03AA |
| Estrogens | NLM:ATC:L02AA |
| Progestogens and estrogens, sequential preparations | NLM:ATC:G03AB |
| Synthetic estrogens, plain | NLM:ATC:G03CB |
| Other estrogens | NLM:ATC:G03CX |
| Selective estrogen receptor modulators | NLM:ATC:G03XC |
| ESTROGENS | NLM:ATC:G03C |
| ESTROGENS | NLM:VA:HS300 |
| ESTRADIOL CREAM, Vaginal (VAG) | NLM:VA:GU500 |
| Progress outcome | |
| Death | R99 |
| Sepsis | A41; R65.2; A40.8; A40.9 |
| Hypertension | I10 |
| Cerebral infarction | I63 |
| Cardiac infarction | I21; I22 |
| acute kidney failure (AKF) | N17 |
| CKD | N18 |
| Osteoporosis | M80; M81 |
| Breast cancer | C50 |
| Endometrosis | N85.0 |
| Endometrium malignancy | C54.1; C54.2 |
| Hypertrophic scar | L91 |
| Skin wound infection | L08 |
UMLS:ICD10CM ; or

## Slide 2
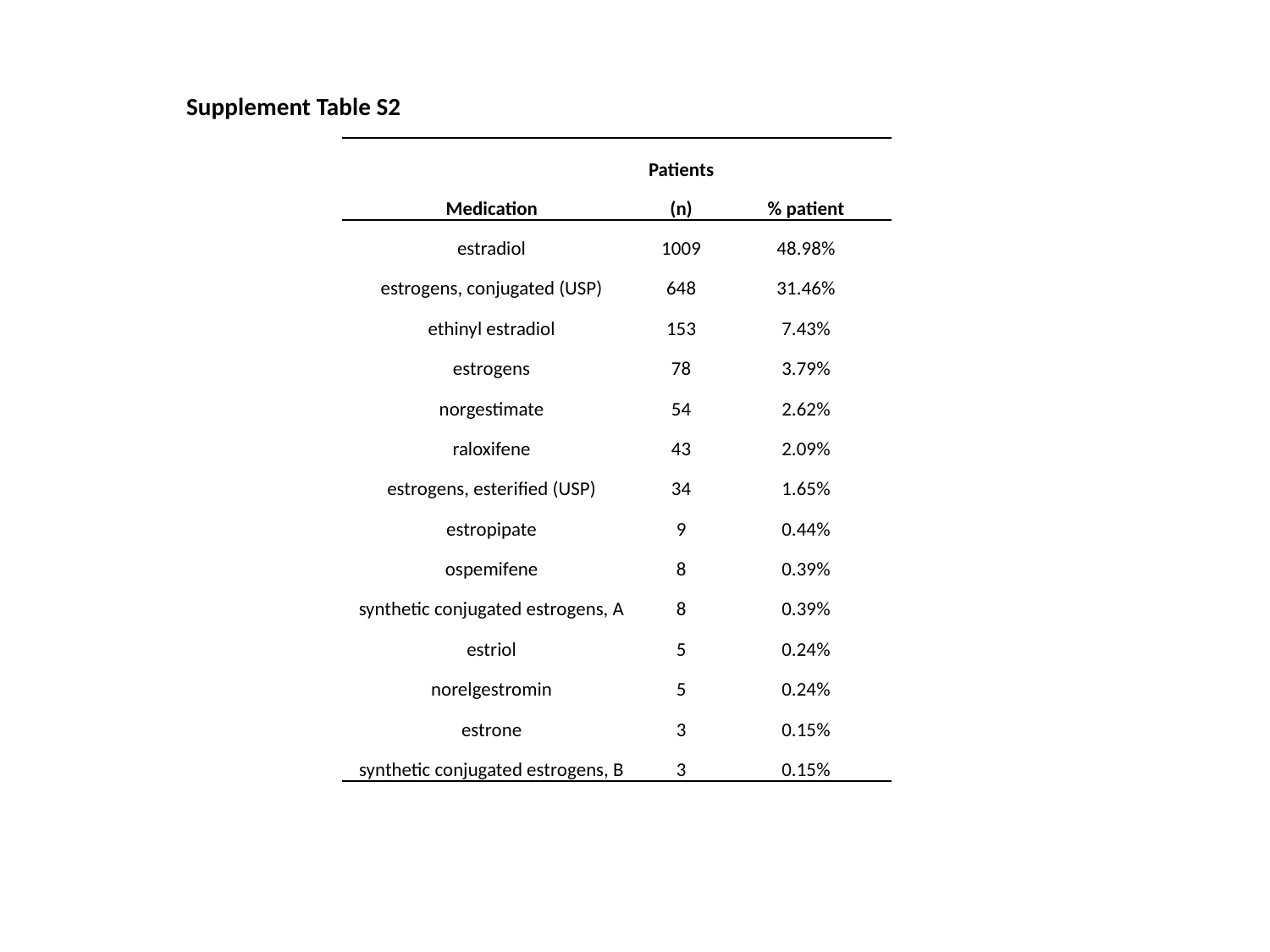

Supplement Table S2
| Medication | Patients (n) | % patient |
| --- | --- | --- |
| estradiol | 1009 | 48.98% |
| estrogens, conjugated (USP) | 648 | 31.46% |
| ethinyl estradiol | 153 | 7.43% |
| estrogens | 78 | 3.79% |
| norgestimate | 54 | 2.62% |
| raloxifene | 43 | 2.09% |
| estrogens, esterified (USP) | 34 | 1.65% |
| estropipate | 9 | 0.44% |
| ospemifene | 8 | 0.39% |
| synthetic conjugated estrogens, A | 8 | 0.39% |
| estriol | 5 | 0.24% |
| norelgestromin | 5 | 0.24% |
| estrone | 3 | 0.15% |
| synthetic conjugated estrogens, B | 3 | 0.15% |

## Slide 3
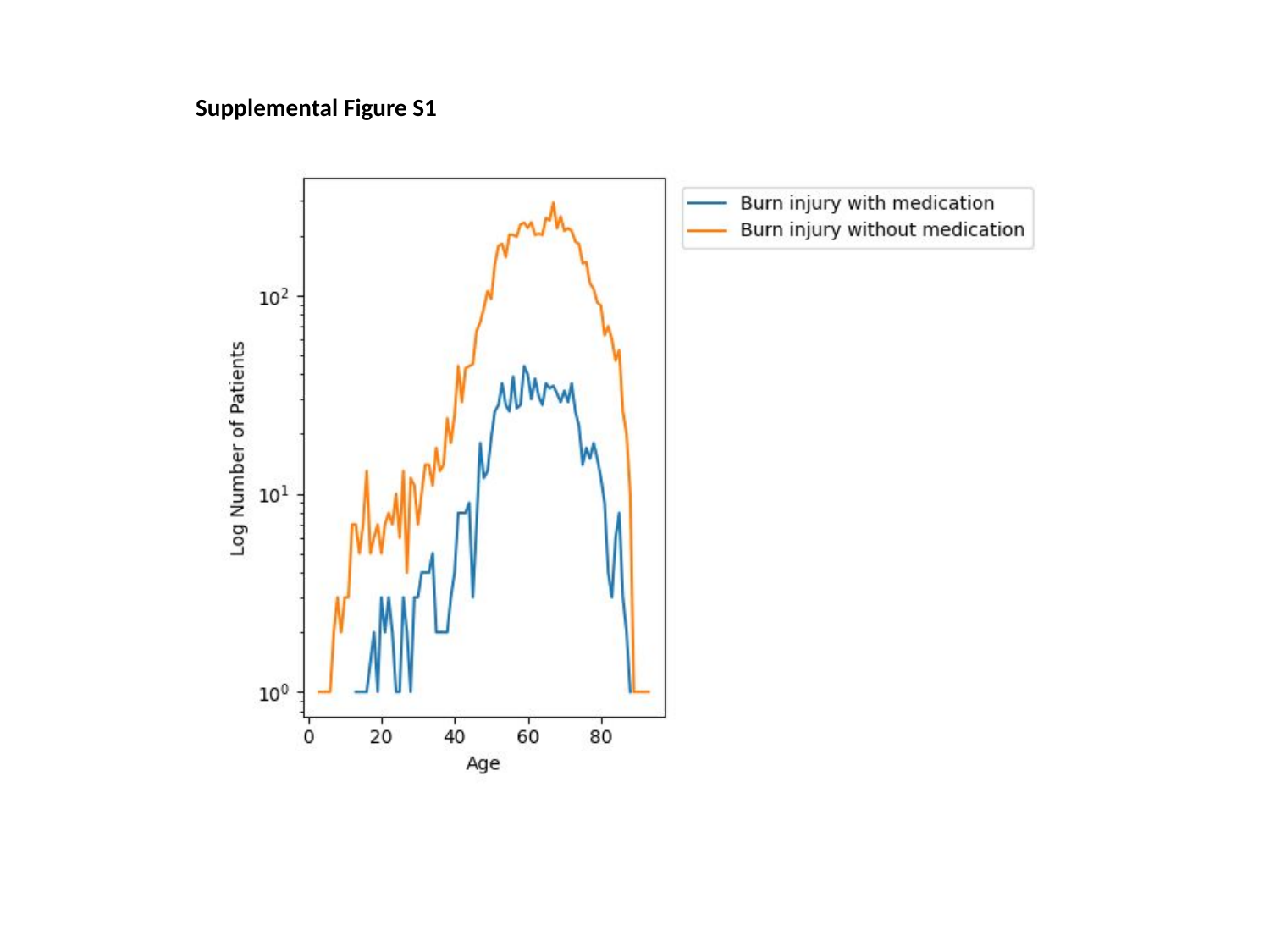

Supplemental Figure S1
